# Origins of Avian Hyperactive Mitochondria, Genome Compaction, and Air-Sac Physiology in Early Theropods During the Carnian Pluvial Episode

**DOI:** 10.3390/jdb14010011

**Published:** 2026-03-02

**Authors:** Takumi Satoh

**Affiliations:** Department of Antiaging Food Research, School of Bioscience and Biotechnology, Tokyo University of Technology, 1404-1 Katakura, Hachioji 192-0982, Japan; satotkm@stf.teu.ac.jp

**Keywords:** super-mitochondria, PT boundary, birds, theropods, insulin, glucagon, air sac

## Abstract

Extant birds and the earliest dinosaurs may share fundamental metabolic features essential for aerobic exercise, suggesting that the extraordinary physical performance typical of avian species originated when dinosaurs first appeared during the Carnian Pluvial Episode (CPE). This physiological adaptation is complemented by hyperactive mitochondria that exhibit high oxygen consumption and low reactive oxygen species production. Molecular genomics of fossils, the so-called “Jurassic Genome,” indicates that these early dinosaurs possessed compact genomes, 50–60% the size of the human genome, and small cells, implying a highly stringent metabolic regime. We suggest that hyperactive mitochondria, closely associated with compact genomes and small cells, drive theropod adaptation to the hot, dry, and hypoxic environments of the Late Triassic period, ultimately enabling their ecological dominance. Early dinosaurs such as Herrerasaurus are hypothesized to have possessed advanced physiological traits shared with modern birds, including hyperactive mitochondria, compact genomes, small cells, and a developing air-sac system. Collectively, these features most likely may have contributed to exceptional metabolic capacity, locomotor performance, and adaptation to the harsh environment of the CPE.

## 1. Introduction

Given that direct evidence of mitochondrial metabolism is not preserved in the fossil record, the timing and mechanisms by which avian-type hyperactive mitochondria and associated high physical performance were established in the theropod–bird lineage remain unclear. This review addresses these questions by proposing a working hypothesis integrating avian physiology, cell biology, and ecology. Birds exhibit this feature because their bodies are uniquely adapted for sustained flight and endurance, with a respiratory system optimized to support exceptionally high aerobic activity, even at extreme altitudes [1,2]. Such mitochondrial activity may be maintained by a unique balance in the insulin–glucagon axis (IGA). Birds possess constitutively active glucagon receptors and exhibit marked insulin resistance, maintaining mitochondria in an intrinsically active state [1,2]. We propose that these traits were inherited from the earliest dinosaurs, such as *Herrerasaurus* and *Coelophysis*, during the Triassic Period. Notably, the earliest dinosaurs emerged during the hot and humid conditions of the Carnian Pluvial Episode (CPE). They exhibited athletic bone structures, a primitive air-sac system, and fully developed bipedal locomotion, enabling strenuous activity even under low-oxygen conditions. These physiological and anatomical characteristics are best explained by evolutionary adaptations in aerobic metabolism, including hyperactive mitochondria and an efficient lung–air sac gas exchange system. Under the harsh environment of the CPE, theropod mitochondria may have been exceptionally active [3,4]. We suggest that hyperactive mitochondria, closely associated with compact genomes and small cells, drove theropod adaptation to the hot, dry, and hypoxic environments of the Late Triassic period, ultimately enabling the later evolution of powered flight during the oxygen-enriched conditions of the late Jurassic [3,4].

## 2. Avian Physical Performance

Among terrestrial animals, birds are considered “extreme athletes,” exhibiting exceptional endurance, speed, and strength. For example, bar-headed geese can fly over the Himalayas at altitudes where oxygen availability is extremely low. This feat is achieved through a highly efficient gas exchange system, enhanced oxygen transport, and remarkable aerobic capacity, which synergistically enable sustained vigorous exercise in hypoxic environments [5,6,7,8,9,10]. Birds possess a flow-through lung system with air sacs that enables continuous oxygen exchange, in contrast to the tidal breathing pattern observed in mammals. This respiratory architecture confers superior gas-exchange efficiency and supports prolonged activity even under oxygen-poor conditions [11,12,13,14,15,16,17].

Additionally, avian muscles and other tissues exhibit enhanced oxygen diffusion capacity, enabling rapid oxygen delivery to working muscles. This adaptation supports both long-distance migration and short bursts of intensely flight [11,12,13]. Birds also maintain elevated circulating levels of glucose and ketone bodies, ensuring a stable and flexible energy supply. Consequently, their mitochondria operate at a high metabolic rate fueled by dual energy substrates, supporting endurance while minimizing oxidative stress. This metabolic strategy ensures the continuous availability of carbohydrates and lipids as fuel sources [14,15,16,17].

## 3. Avian and Mammalian Lungs

**Figure 1:**
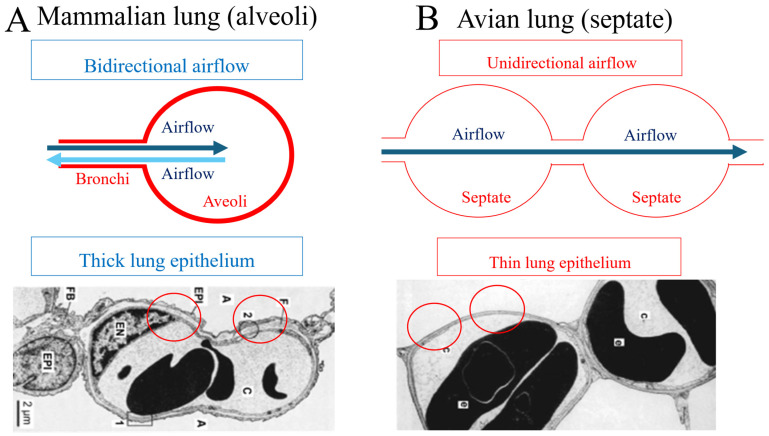
Comparison of mammalian and avian lung structures and airflows. (**A**) Mammalian lungs. Mammals exhibit bidirectional (tidal) airflow, in which air moves in and out of the lungs along the same pathway. Therefore, the fresh inhaled air mixes with residual air, reducing effective oxygen concentrations. Additionally, the mammalian lung epithelium is relatively thick, limiting gas-exchange efficiency [8,9,10,11,12,13]. (**B**) Avian lungs. Birds possess a unidirectional airflow system, in which air passes through the lungs in a single direction, enabling more efficient oxygen extraction. The avian lung epithelium is extremely thin, markedly enhancing gas-exchange capacity. Red circles indicate the lung epithelium [17,18,19]. Images are reproduced from [17]. Electron micrograph of blood capillaries from a mammal (dog) and a bird (chicken) lung. Circles in the photographs show the thickness of lung epithelium of mammals and birds. A: air capillary; C: blood capillary; EN: endothelial cells; EPI: lung epithelium; FB: fibroblast.

As mentioned earlier, the most fundamental feature of the avian gas exchange system, which constitutes them as “extreme athletes,” is that air flows unidirectionally through the lungs and air sacs, enabling high-efficiency oxygen uptake even under hypoxic conditions [14,15,16]. Electron microscopy reveals that the avian lung epithelium is markedly thinner than that of mammals [17,18,19]. Birds possess septate lungs, a structural specialization permitting extreme thinning of the pulmonary epithelium. This adaptation significantly enhances gas-exchange efficiency by facilitating the rapid diffusion of oxygen and carbon dioxide across the respiratory interface. John B. West identified epithelial thinning as the key mechanism underlying this difference [17,18,19] (Figure 1).

## 4. Insulin–Glucagon Axis (IGA)

**Figure 2:**
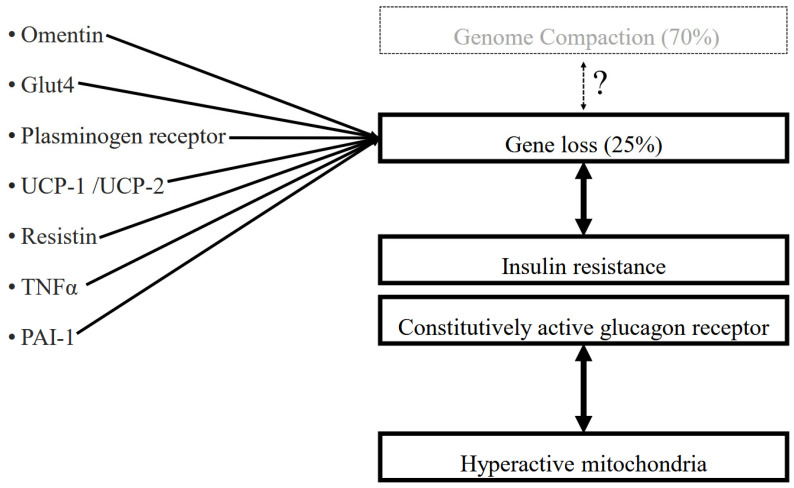
Hyperactive mitochondria regulated by the IGA. Gene loss (omentin, Glut4, plasminogen receptor, UCP-1/UCP-2, Resistin, TNFα, and PAI-1) and mutation contribute to insulin resistance [1,2] and enhanced glucagon signaling [23,24,25], collectively promoting a constitutively active metabolic state and the emergence of hyperactive mitochondria. It is highly possible that these genes were lost during the CPE during genome compaction, although no direct evidence demonstrating that genome compaction is causally linked to gene loss.

The IGA provides a critical framework for understanding the hormonal regulation of avian physical activity, functioning differently from that in mammals. Whereas mammalian metabolism tightly relies on insulin-mediated regulation of blood glucose and ketone bodies, birds have evolved a glucagon-dominated system that supports a hyperactive lifestyle and enhanced physical performance [20,21,22] (Figure 2). This mechanism favors circulating glucose availability over glycogen storage, ensuring immediate energy supply to meet elevated metabolic demands [23,24,25]. Birds also maintain elevated circulating levels of glucose and ketone bodies, providing a stable and flexible energy supply. Consequently, their mitochondria operate at high metabolic rates fueled by dual energy substrates, ensuring continuous availability of carbohydrates and lipids as energy sources [26,27,28]. Furthermore, birds exhibit markedly elevated circulating glucagon levels compared with mammals [26,27,28] (Table 1).

The striking feature of extant birds are significant insulin resistance [29,30,31]. The avian genome contains approximately 15,000 protein-coding genes, whereas mammalian genomes typically contain >20,000 genes [32,33,34]. Approximately 25% of genes have been lost in extant birds. Several genes essential for insulin sensitivity have been lost in birds [35,36,37,38]. It is suggested that this gene loss contributes to insulin resistance. In birds [1], the insulin signaling pathway appears markedly attenuated in adipose tissue and skeletal muscle, as indicated by reduced insulin receptor phosphorylation, although insulin is retained in the liver. Insulin resistance in adipose tissue and skeletal muscle of chickens may activate mitochondrial metabolism and increase oxygen consumption [39,40,41,42,43,44]. Insulin is essential for embryonic development in both mammals and birds [45,46]. Mammals retain insulin sensitivity throughout life death, whereas birds become insulin-resistant early in life [47].

Given that birds possess a constitutively active glucagon receptor, glucagon signaling remains persistently engaged, thereby driving sustained hepatic glucose release and chronically elevated blood glucose levels. Glucagon promotes energy mobilization by stimulating gluconeogenesis and mitochondrial respiration while suppressing cell growth and proliferation. It also activates the peroxisome proliferator-activated receptor-γ coactivator-1α, a central regulator of mitochondrial biogenesis, thereby enhancing aerobic capacity [23,24,25,26,27,28,29,30,31,32,33,34,35].

## 5. Avian and Mammalian Mitochondria

Hyperactive avian mitochondria consume large amounts of oxygen while producing minimal levels of reactive oxygen species (ROS). They actively eliminate ROS, thereby limiting oxidative damage, slowing aging-related processes, and supporting longevity. Avian cells are densely populated with mitochondria, enabling large-scale energy production and ROS elimination. During prolonged activity, birds steadily increase oxygen consumption while maintaining extremely low ROS release levels [48,49,50] (Table 2).

**Figure 3:**
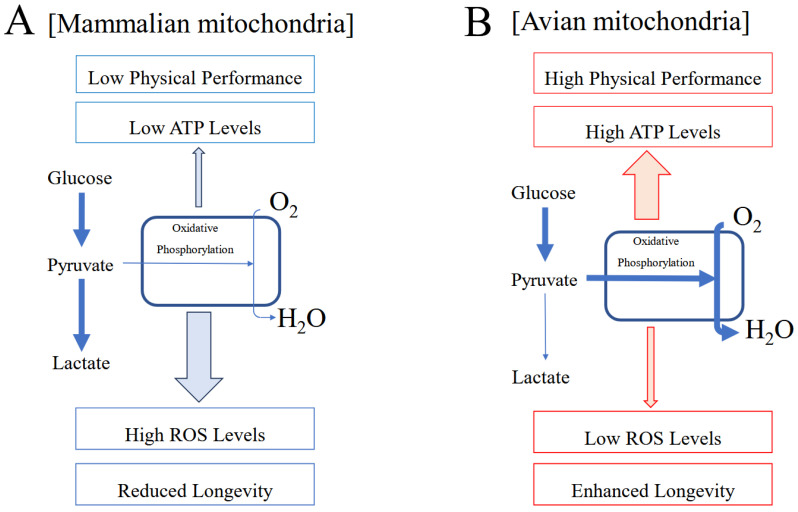
Functional contrasts between mammalian and avian mitochondria. (**A**) Mammalian mitochondria. Under hypoxic conditions, mammals appear to adopt a metabolic strategy that limits oxygen consumption to reduce ROS leakage, thereby suppressing the rate of metabolism. Mammalian mitochondria generate relatively high ROS levels and lower ATP output, a profile associated with reduced longevity and lower sustained physical performance [1,2]. (**B**) Avian mitochondria. Conversely, birds respond to hypoxia by increasing oxygen consumption, maximizing oxygen-utilization efficiency. Avian mitochondria produce minimal levels of ROS while generating high ATP output, a combination associated with extended longevity and superior physical performance [1,2]. Images are reproduced from [1].

Conversely, increased oxygen utilization during intense exercise in mammals enhances ROS production. Therefore, mammalian mitochondria can be characterized primarily as ROS-generating systems. During sustained high-intensity activity, mammalian mitochondria contribute to oxidative stress, thereby accelerating aging [48,49,50] (Figure 3).

## 6. Theropod–Bird Lineage and the Triassic Environment

The PT boundary: Fluctuations in atmospheric oxygen concentrations were among the fundamental drivers of vertebrate evolution. Particularly, the sharp decline in oxygen levels at the PT boundary approximately 252 million years ago (myr) likely represented a severe physiological challenge faced by vertebrates. These hypoxic conditions exerted strong selective pressure on theropods, favoring smaller genomes to maximize oxygen utilization efficiency. The PT boundary marked a profound shift in the body structure of the theropod–bird lineage, facilitating adaptation to persistently thin air. This reorganization likely included the evolution of more efficient gas exchange systems, enabling survival under hypoxia and permitting diversification and ecological dominance during the Triassic period [54,55,56,57,58,59,60,61,62].

Before CPE: The Earth experienced five major mass extinction events, the largest occurring at the PT boundary and closely associated with a dramatic drop in atmospheric oxygen [54,55,56,57,58,59,60,61,62]. This event led to the extinction of more than 95% of all species, mainly due to hypoxia in the early Triassic period [54,55,56,57,58,59,60,61,62]. The surviving vertebrate lineages were subjected to extreme selection pressure, requiring rapid physiological and anatomical adaptations to hypoxic conditions [63,64,65].

**Figure 4:**
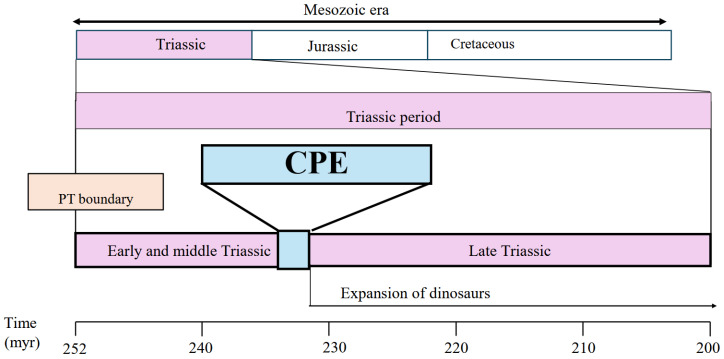
Triassic climate dynamics and dinosaur evolution. During the CPE, early dinosaurs may have rapidly risen to constitute more than 90% of terrestrial vertebrate fauna from 5% as suggested by fossil and footprint evidence [4].

During CPE: The CPE (244–242 myr) represents a remarkable Late Triassic climatic episode characterized by intense global rainfall and elevated humidity that temporarily interrupted the prevailing hot and arid Triassic climate [66,67,68,69,70,71]. During the CPE, dinosaurs’ expansion should have been accompanied by a profound reorganization of body structures, including the establishment of obligate bipedal locomotion, the development of a primitive air-sac system, the compaction of genomes, and the reduction in cell size [1,2,3,4] (Figure 4).

After CPE: In the Late Triassic period, global climate conditions reverted to hot and dry states, resulting in significant ecological restructuring [66,67,68,69,70,71]. Fern-dominated vegetation declined under increasing aridity, whereas drought-resistant conifers persisted and eventually dominated terrestrial ecosystems. Many forests most likely developed open understory spaces beneath tall conifer canopies [72,73,74]. These open habitats favored large, fast-moving theropods, enabling them to capitalize on physiological and locomotor advantages and achieve ecological dominance. At approximately 231 myr, more evolved athletic theropods, such as *Herrerasaurus,* emerged and became the dominant terrestrial predators because of their physical performance [72,73,74].

## 7. Compact Genome of the Earliest Dinosaurs

**Figure 5:**
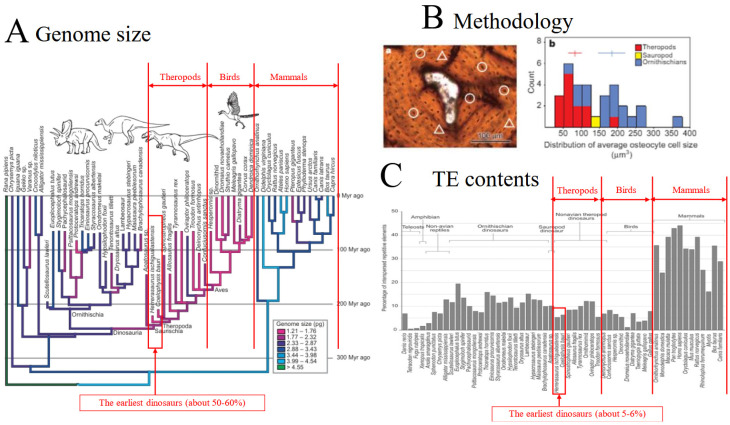
Genome compaction and TE reduction inferred from the Jurassic Genome. (**A**) Genome size comparison. (**B**) Methodology. (**C**) TE contents. When ranked by genome size, the order is mammals > bats > pterosaurs > theropods > birds. Using the human genome, approximately 3.5 billion base pairs’ long, as a reference (defined as 100%), avian genomes are approximately 33% smaller. Notably, early theropods such as *Herrerasaurus* and *Coelophysis* possessed genomes estimated to be 50–60% smaller than the human genome [3]. The authors calculated osteocyte size directly from histological sections of bone by measuring the small pockets in the mineral matrix. Circles indicate the type of cells that were measured and triangles indicate the type of cells that were not measured (**B**).

The PT boundary caused a complete change in the basic vertebrate body plan, facilitating adaptation to hypoxia. This novel body plan may have increased survival probability under hypoxic conditions. Genome compaction is considered a highly effective driver of this transition. Under hypoxia, theropods may have totally changed their body plan by reducing genome size. Genome reduction was demonstrated by the Jurassic genome. The genome sizes of extinct dinosaurs can be inferred from fossilized cell dimensions, particularly osteocyte lacunae. Chris Organ demonstrated a progressive decrease in genome size along the avian lineage—from diapsids to small theropods and ultimately to Neoaves—suggesting that genome compaction played a central role in theropod and avian evolution [3,75,76,77,78]. One plausible mechanism underlying genome compaction is the suppression of TEs [79,80,81,82]. Given that TEs significantly contribute to the maintenance and expansion of the vertebrate genome size, reduced TE abundance promotes genome compaction [79,80,81,82]. TEs comprise 30–40% of mammalian genomes, approximately 10% of theropod genomes, and 2–3% of avian genomes, indicating a close association between progressive TE reduction and the evolution of compact genomes [3] (Figure 5).

## 8. Emergence of Hyperactive Mitochondria

**Figure 6:**
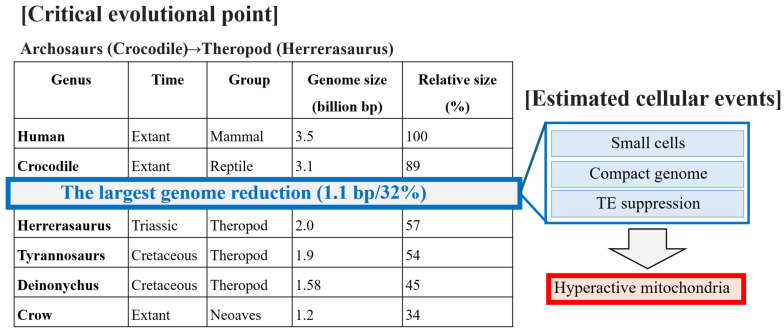
CPE as a possible critical point leading to genome compaction and hyperactive mitochondria. The greatest genome reduction within a very short time [250 (PT boundary)–231 (Herrerasaurus) myr] may have occurred when the total change in body plan became dedicated to an adaptation to hypoxia.

Genome compaction may have begun across the PT boundary when theropod lineages diverged from reptiles (crocodiles) and may have recurred during evolution from theropods (*Herrerasaurus*, *Tyrannosaurus*, and *Deinonychus*) to Neoaves (crow). The positive feedback loop between small cells, compact genomes, and TE suppression driven by hypoxia may have lasted for approximately 20 million years, up to the onset of the CPE [54,55,56,57,58,59,60,61,62]. Notably, the earliest dinosaurs, which appeared just after the CPE [4], possessed genomes approximately 50% the size of the human genome [3], consistent with pronounced genome compaction [83,84,85,86,87]. These traits may have been closely linked to increased metabolic activity and may underline the development of hyperactive mitochondria [83,84,85,86,87]. Particularly, dinosaur expansion during the CPE increased from 5% to 90% [4]. Therefore, the estimated timing of compact genome emergence, TE suppression, and small-cell evolution may be concentrated within the relatively short CPE interval (234–232 myr). Additionally, just after the CPE, the first theropod, *Herrerasaurus*, which is supposed to possess hyperactive mitochondria, appeared (Figure 6).

Another proxy for mitochondria activation may allow for direct reconstruction of metabolic rates from fossils. In situ Raman and Fourier-transform infrared spectroscopy can quantify the in vivo accumulation of metabolic lipoxidation signals in modern and fossil amniote bones. Inferred ancestral states reveal that theropods had high metabolic rates. Because these signals originated from mitochondria, the suggest that theropods possessed more active mitochondria than other terrestrial animals [88]. However, given that this lipoxidation research focused on Jurassic and Cretaceous theropods, there is no direct evidence on the Triassic period [88]. Furthermore, the Jurassic genome provides evidence of compact genomes only from *Herrerasaurus* and *Coelophysis* among Triassic theropods [3]. Further research on the earliest theropods and evidence of hyperactive mitochondria is required.

## 9. How Did Hypoxia Lead to Avian Hyperactive Mitochondria?

**Figure 7:**
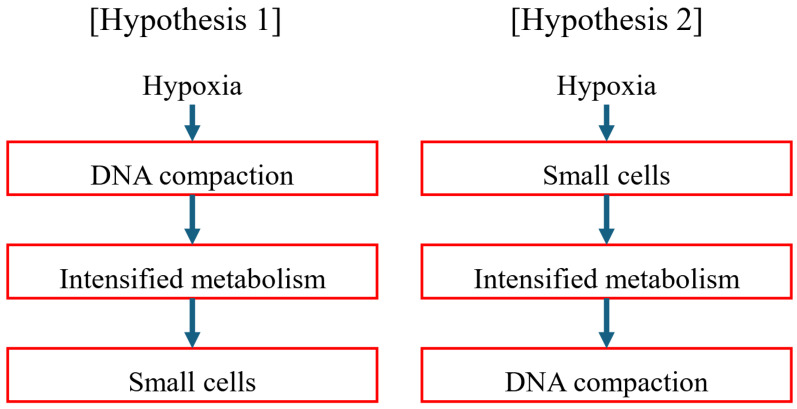
There may be two possible hypotheses regarding how the positive feedback loop was initiated. One hypothesis is based on molecular biology, and the other is based on cellular physiology.

A possible pathway for the emergence of hyperactive mitochondria may have been initiated by a positive feedback loop among compact genome [86,87,88,89], intensified metabolism [48,49,50], and small cells [83,84,85], ultimately leading to the emergence of hyperactive mitochondria [1,2]. How was this positive feedback loop initiated in the theropod lineage? The premise is that hypoxia must have been the most decisive evolutionary driver during the Triassic period. (Figure 7).

Genome DNA may harbor genes that suppress aerobic mitochondrial metabolism, possibly via insulin signaling molecules. In other words, cells harbor genes that regulate mitochondrial activity to prevent dysregulation [35,36,37,38]. If these genes were lost during genome compaction, as shown in Figure 2 [32,33,34], metabolism may have become intensified [1,2]. Increased energy substrates per cell volume would need to be taken up through the introduction of smaller cells [83,84,85], further intensifying metabolism [48,49,50].

Alternatively, in response to hypoxia, cells may increase oxygen and energy substrate uptake per cell volume through cell membrane by reducing cell size [83,84,85]. Increased oxygen uptake may intensify metabolism [1,2]. Cells may allocate available resources toward metabolism by reducing DNA content, ultimately leading to genome compaction [32,33,34].

These causal relationships may become circular and may collectively lead to the emergence of hyperactive mitochondria [1,2]. Hypothesis 2 may be more likely, as increased oxygen supply per volume could occur rapidly. In contrast, hypothesis 1 may require additional time to activate metabolism in response to sudden hypoxia.

## 10. CPE as the Final Boost for Dinosaurs’ Expansion

The final major expansion of dinosaurs was likely accelerated by the CPE (234–232 myr), a short but intense climatic disruption which occurred during the Triassic period. The earliest known theropod, *Herrerasaurus*, appeared thereafter (approximately 231 myr). By this interval, theropods had already adapted to hypoxic conditions and undergone substantial genome reduction, which played a pivotal role in reshaping body structure and enhancing physiological performance [64,65,66].

Geological and sedimentological evidence indicates widespread monsoon-like rainfall and regional flooding during the CPE. This event was likely triggered by extensive volcanic activity in the Wrangellia Large Igneous Province, releasing massive quantities of CO_2_ and other gases into the atmosphere. The resulting greenhouse warming intensified evaporation and strengthened global hydrological cycles [67,68,69,70,71].

Early theropods such as *Herrerasaurus* had emerged, and the number of dinosaurs rapidly increased to constitute more than 90% of terrestrial vertebrate fauna, as suggested by fossil evidence. This expansion was accompanied by a significant reorganization of body structures, including the establishment of obligate bipedal locomotion, the development of a primitive air-sac system, the compaction of genomes, and a reduction in cell size [72,73,74,75].

The Triassic period was characterized by low atmospheric oxygen levels and extreme heat. Sustained locomotion under such conditions requires highly efficient heat-dissipation mechanisms. In theropods and modern birds, air sacs extended into major skeletal elements and internal cavities, forming an extensive internal ventilation network that likely provided a crucial adaptive advantage during the intense Triassic climate [89,90,91,92,93,94].

Faster movement enhances the effectiveness of this internal cooling system. In theropods and birds, air sacs permeate much of the body, enabling direct airflow from the environment. During sustained high-speed locomotion, this system allows uniform body cooling at wind speeds comparable to movement velocity. This adaptation enabled theropods to maintain rapid locomotion under extreme heat without succumbing to heat stress, conferring exceptional thermal resilience [89,90,91].

**Figure 8:**
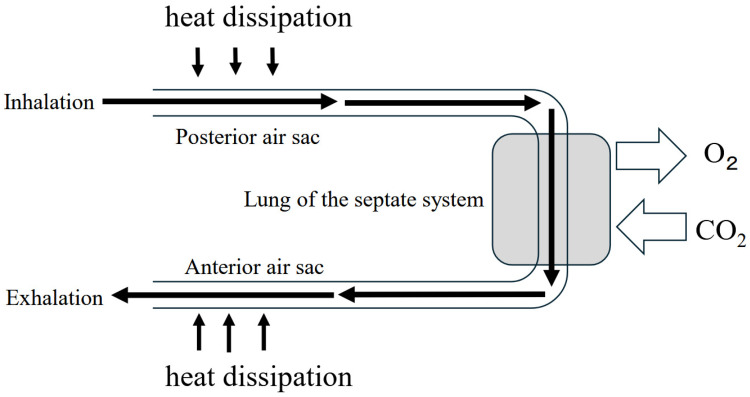
Dual functions of air sacs in gas exchange and heat dissipation. Theropods may have evolved hollow bones that house pulmonary extensions known as air sacs. Beyond enhancing respiratory efficiency, air sacs likely played a decisive role in heat dissipation [92,93,94].

The evolution of air sacs may therefore be of particular significance [89,90,91]. By extending the pulmonary structures into large, hollow bones, this system greatly enhanced ventilatory capacity and oxygen delivery. Heat stress during the hot and humid CPE conditions may have been more severe than during other Triassic intervals. High humidity substantially amplifies thermal stress in terrestrial animals. In theropods, the air-sac system may have functioned to enhance gas exchange and improve heat tolerance. This physiological adaptation may have contributed substantially to dinosaurs’ dominance over other terrestrial vertebrates during the CPE [92,93,94] (Figure 8).

During prolonged locomotion, air flows rapidly through the lungs, continuously supplying oxygen at elevated levels. Increased velocity may further accelerate internal airflow, thereby improving gas exchange and heat dissipation. These physiological advantages may, in turn, promote skeletal and muscular adaptations supporting high speed locomotion [1]. Such adaptations may have enabled theropods to sustain rapid locomotion under extreme heat without succumbing to heat stress, conferring an exceptional cooling system [92,93,94].

## 11. New Avenues and Limitations of the Present Study

This study opens two new conceptual avenues. First, it proposes that the hyperactive avian mitochondria originated in the earliest dinosaurs. Second, it advances the hypothesis that the air-sac system evolved to enhance gas exchange and confer heat-stress resistance. Together, these perspectives provide a unified physiological framework for understanding dinosaur and avian success under extreme environmental conditions.

**Figure 9:**
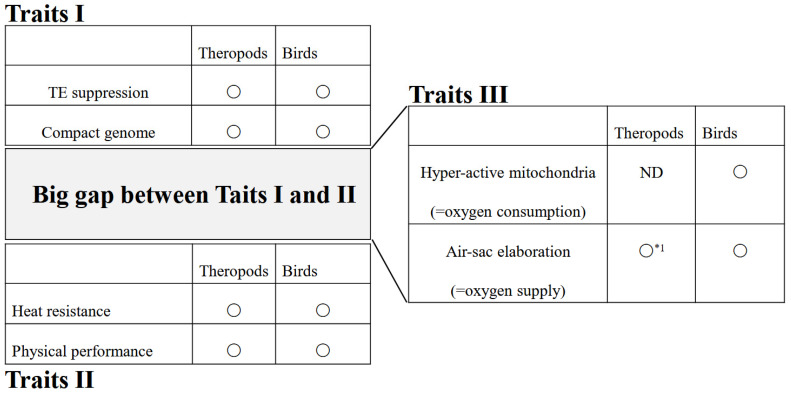
Hyperactive mitochondria and air-sac elaboration. Traits I (genome level) and II (framework level) are largely established theories, yet a significant gap remains between them. Traits III (cellular or organ levels), including hyperactive mitochondria and air-sac elaboration, may provide an effective bridge across this gap. 〇: admitted as an established theory; 〇*^1^: air-sac elaboration in the earliest dinosaurs has not been precisely examined, although many researchers agree on functional air sacs; ND: not determined by fissile examination.

This study is inherently interdisciplinary, integrating evidence from physiology, molecular biology, paleontology, and ecology. Consequently, it is limited by the absence of direct fossil evidence supporting physiological and molecular traits. Parameters such as mitochondrial activity, hormonal regulation, and cellular metabolism are not directly preserved in the fossil record. Therefore, the hypotheses presented here rely only on indirect inference and comparative biological reasoning rather than direct observation (Figure 9).

## 12. Conclusions

The extraordinary physical performance of birds originates from a unique mitochondrial phenotype, often referred to as “super mitochondria,” characterized by exceptionally high metabolic performance [1]. These mitochondria, which likely played a central role in the evolutionary transition from theropods to birds, are hypothesized to have emerged in organisms adapted to chronical hypoxic environments [1]. This evolutionary transformation was particularly pronounced during the Triassic period, when theropods diverged from their diapsid ancestors under atmospheric oxygen levels lower than those prevailing during most other intervals in Earth’s history. During this interval, theropods underwent extensive genomic reorganization and profound restructuring of their body plans. These changes coincided with the emergence of super mitochondria, enabling the development of exceptional locomotor capacity. During the CPE, the combined effects of hyperactive mitochondria and the air-sac system—functioning in both gas exchange and heat dissipation—likely conferred a decisive adaptive advantage. Together, these innovations contributed to the ecological dominance of dinosaurs and laid the physiological foundation for the later success of birds.

## 13. Methodological Framework

This study is inherently interdisciplinary, integrating evidence from physiology, molecular biology, paleontology, and ecology. It is limited by the absence of direct fossil evidence supporting physiological and molecular traits, as parameters such as mitochondrial activity, hormonal regulation, and cellular metabolism are not directly preserved in the fossil record. Consequently, the hypotheses presented in this study rely on indirect inference and comparative biological reasoning rather than direct observation. The methodological backbone is therefore implicit and distributed across sections, most clearly in: (i) comparative physiological analyses of avian versus mammalian lungs and mitochondria (Section 1, Section 2 and Section 3 [1,2]); (ii) indirect genomic inference using osteocyte lacuna size as a proxy for genome size (“Jurassic Genome”; Section 5 and Section 6), and (iii) integration of paleoclimate reconstructions of atmospheric oxygen and the Carnian Pluvial Event (Section 7). In Figure 5, genome size and interspersed repetitive element content were inferred using primary histological data, including osteocyte size distribution in extinct dinosaur species, combined with regression models calibrated using extant taxa [3]. In addition, the genomes of theropods and birds exhibit substantially reduced transposable elements (TE) content compared with mammals, consistent with progressive genome compaction during theropod and avian evolution [3]. Data of Figure 5 are derived from [3].

## Figures and Tables

**Table 1:** Energy substrates and hormones in the blood. Due to insulin resistance and a constitutive-active glucagon receptor, the levels of both glucose and ketone bodies are significantly elevated in birds. Additionally, insulin levels are lower and glucagon levels are higher in birds, possibly supporting enhanced physical performance.

	Mammals	Birds	Refs
Glucose (mg/dL)	80–100	300–400	[26]
Ketone bodies (mg/dL)	1–2	10–25	[26]
Insulin (pmol/L)	14–700	7–210	[27]
Glucagon (pg/mL)	20–50	300–900	[28]

**Table 2:** Features of mammalian and avian mitochondria: Mammalian and avian mitochondria exhibit different performance profiles. Avian mitochondria demonstrate (i) high oxygen consumption capacity, (ii) strong resistance to oxidative damage, and (iii) high mitochondrial density.

	Mammals	Birds	Refs
O_2_ consumption (pmol/min/2000 cells)	30–35	60–80	[51]
Oxidative damage (mM H_2_O_2_ equivalent)	23–28	5–8	[51]
Mitochondrial density per muscle cell (%)	8–10	40–50	[52,53]

## Data Availability

No datasets were generated or analyzed in this current review. But data will be made available on reasonable request.

## Abbreviations

CPE	Carnian Pluvial Episode
IGA	insulin–glucagon axis
myr	million years ago
PT boundary	Permian–Triassic boundary
ROS	reactive oxygen species
TE	transposable element
